# Metallic edge states in zig-zag vertically-oriented MoS_2_ nanowalls

**DOI:** 10.1038/s41598-019-52119-3

**Published:** 2019-10-30

**Authors:** Miguel Tinoco, Louis Maduro, Sonia Conesa-Boj

**Affiliations:** 1 Kavli Institute of Nanoscience, Delft University of Technology, 2628CJ Delft, The Netherlands; 2 ICTS – Centro Nacional de Microscopía Electrónica, Universidad Complutense, 28040 Madrid, Spain

**Keywords:** Electronic properties and materials, Structural properties, Two-dimensional materials

## Abstract

The remarkable properties of layered materials such as MoS_2_ strongly depend on their dimensionality. Beyond manipulating their dimensions, it has been predicted that the electronic properties of MoS_2_ can also be tailored by carefully selecting the type of edge sites exposed. However, achieving full control over the type of exposed edge sites while simultaneously modifying the dimensionality of the nanostructures is highly challenging. Here we adopt a top-down approach based on focus ion beam in order to selectively pattern the exposed edge sites. This strategy allows us to select either the armchair (AC) or the zig-zag (ZZ) edges in the MoS_2_ nanostructures, as confirmed by high-resolution transmission electron microscopy measurements. The edge-type dependence of the local electronic properties in these MoS_2_ nanostructures is studied by means of electron energy-loss spectroscopy measurements. This way, we demonstrate that the ZZ-MoS_2_ nanostructures exhibit clear fingerprints of their predicted metallic character. Our results pave the way towards novel approaches for the design and fabrication of more complex nanostructures based on MoS_2_ and related layered materials for applications in fields such as electronics, optoelectronics, photovoltaics, and photocatalysts.

## Introduction

The ability of crafting new materials in a way that makes possible controlling and enhancing their properties is one of the main requirements of the ongoing nanotechnology revolution^1,2^. In this context, a family of materials that has attracted intense attention recently are 2D layered materials, such as MoS_2_, which belong to the group of transition metal dichalcogenides (TMDs). These materials have been extensively studied due to their promising electrical and optical properties^3–6^. A defining feature of TMDs is that they exhibit a lack of inversion symmetry, which leads to the appearance of a variety of different edge structures. The most common of these, consisting on dangling bounds, are the armchair (AC) and the zig-zag (ZZ) edge structures.

Of particular relevance in this context, the electronic properties of MoS_2_ have been predicted to be affected by the presence of the different edge structures in rather different ways. For instance, the AC edges have been predicted to be semiconducting, while the ZZ edges should exhibit instead metallic behavior^7–10^. Moreover, *ab-initio* theoretical calculations predict that these metallic states at the edges of MoS_2_ could lead to the formation of plasmons^11^. Beyond this tuning of electronic properties, other attractive applications of these active edge sites arise in photocatalysis, such as their use in hydrogen evolution reactions (HER)^12–15^.

With these motivations, it is clear that the design and fabrication of MoS_2_ nanostructures with morphologies that maximize the number of exposed active edge sites is a key aspect for further improvements in terms of applications. In this respect, significant efforts have been pursued to realize the systematic bottom-up growth of vertically-oriented standing MoS_2_ layers. This configuration leads to the edge sites facing upwards, therefore maximizing the number of exposed edge sites as compared with the more common horizontal configuration, where its basal plane lies parallel to the substrate^16–23^. However, this bottom-up approach is hampered by a lack of reproducibility due to the complexities of the growth mechanism. Another limitation within the bottom-up approach is that the specific type of edges exposed cannot be selectively grown.

Ideally, one would like to combine the best of both worlds. On the one hand, it is important to be able to controllably grow MoS_2_ nanostructures that exhibit the largest possible surface area of edge structures, as it is achieved by the bottom-up strategy summarized above. On the other hand, one would also like to be able to select the specific type of edge sites exposed, in particular, by selecting whether these correspond to AC or to ZZ edges. Therefore, the main goal of this work is to bridge these two requirements by realizing a novel approach to the growth of vertically-oriented standing MoS_2_ layers with full control on the nature of the exposed edge sites.

To achieve this goal, here we adapt a well-stablished top-down approach based on focus ion beam (FIB) in a way that allows us to selectively pattern both types of edges (AC and ZZ) within out-of-plane (vertical) MoS_2_ nanostructures. In the context of patterning layered materials, the usefulness of FIB has been repeatedly demonstrated^24–26^. By means of this technique, we are able to selectively maximize the density of exposed edge sites while controlling their type. Subsequently, by combining high-resolution transmission electron microscopy (TEM) with electron energy-loss spectroscopy (EELS) measurements, we are able to confirm not only the crystallographic nature of both the AC and ZZ MoS_2_ surfaces, but also we can demonstrate that, despite the roughness and imperfections induced during the fabrication procedure, the ZZ MoS_2_ nanostructures clearly exhibit a metallic character, in agreement with the theoretical predictions from *ab-initio* calculations^11^.

The results of this work will open new opportunities for nanoengineering the edge type in MoS_2_ nanostructures as well as in related layered materials, paving the way towards novel exciting opportunities both for fundamental physics and technological applications in electronics, optoelectronics, photovoltaics, and photocatalysts.

## Results

**Figure 1:**
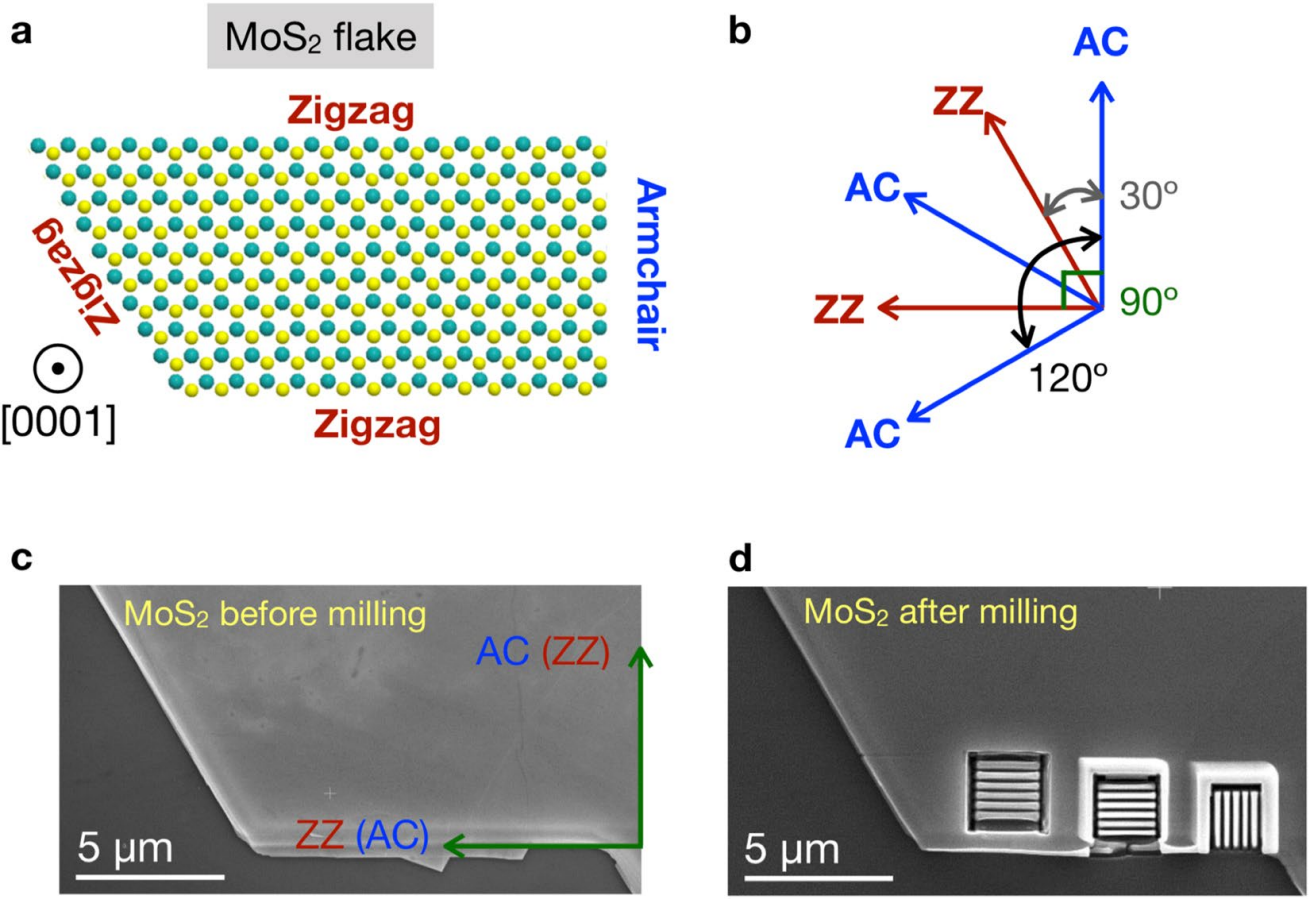
(**a**) Atomic model of a MoS_2_ flake viewed along the [0001] direction, where we indicate the corresponding zig-zag (ZZ) and armchair (AC) edges. **(b)** From geometric considerations, we can determine the possible values that the angles between adjacent AC and ZZ edges should take; **(c)** and **(d)** SEM micrographs of the MoS_2_ flake used for patterning the nanowalls, taken before and after the milling respectively. In **(d)**, three different set of arrays can be observed. The left-most array was fabricated without the protective metal layer, while the other arrays used instead this protective metal layer.

From crystal structure considerations, the possible angles between adjacent flat edges within MoS_2_ flakes should be multiples of 30°. Specifically, the expected angles between adjacent AC and ZZ edge structures in a MoS_2_ flake such as that of Fig. 1a should be 30°, 90°, and 120°, as illustrated in Fig. 1b. Based on this information, we have designed the orientation of the different areas of the MoS_2_ flake that subsequently will be patterned. In this way, we can ensure the full control over the resulting specific edge crystallographic orientation.

Figure 1c,d display a scanning electron microscopy (SEM) image of the MoS_2_ flake that has been used for the fabrication of the nanostructures, taken before and after the milling respectively. Before the milling is performed, a protective metallic layer of tungsten (W) with a thickness of 500 nm was deposited on top of the selected areas of the MoS_2_ flake. Subsequently, we performed a series of milling and cleaning processes in order to construct the vertically-aligned MoS_2_ nanostructures. Figure 1d displays three ordered vertically-oriented patterned arrays of MoS_2_ nanostructures, which in the following are denoted as nanowalls (NWs). Two of these sets of nanowalls are oriented perpendicularly with respect to each other, guaranteeing that this way one of two arrays will correspond to AC (ZZ) NWs while the other array will correspond instead to the complementary ZZ (AC) ones. These NWs are found to exhibit a uniform thickness being (89 ± 5) nm (central array in Fig. 1d) and (68 ± 5) nm (rightmost array in Fig. 1d). Note that the left-most array was fabricated without the protective metal layer. For further details about the optimization of the MoS_2_ nanowalls see the Supplementary Information.

To further examine the crystallographic nature of the resulting vertical MoS_2_ nanostructures, transmission electron microscopy (TEM) studies were carried out. For these studies, we lifted out two of the MoS_2_ NWs from the two different patterned NWs arrays using a micromanipulator. Subsequently, the nanostructure was mounted onto a TEM half-grid. This whole procedure takes place within the FIB chamber.

**Figure 2:**
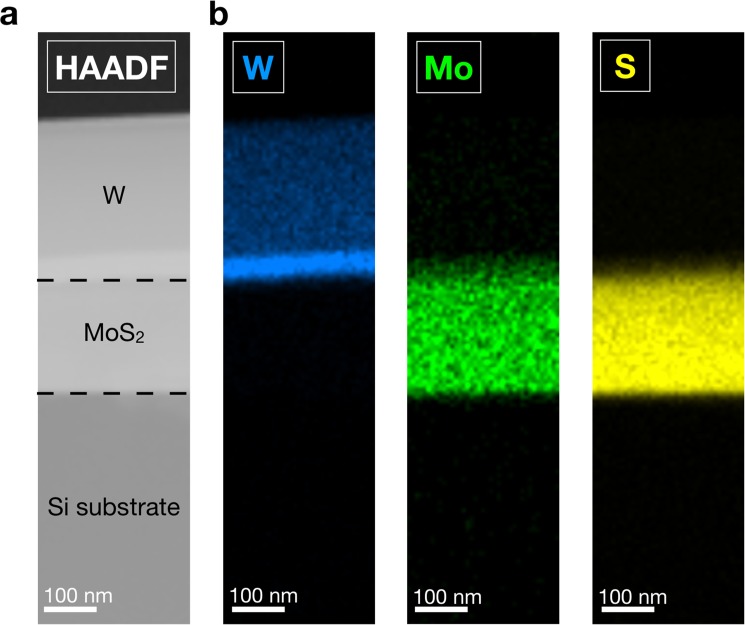
(**a**) HAADF-STEM image of an area of the ZZ MoS_2_ NW, which is bracketed between the Si substrate and the metallic protective layer; (**b**) The corresponding EDX compositional maps (Mo in green, S in yellow, W in blue).

Figure 2a displays a high-angle annular dark-field scanning transmission electron microscopy (HAADF-STEM) image of a selected region of the ZZ MoS_2_ nanowall, bracketed between the Si substrate and the metallic protective layer. Figure 2b shows the corresponding chemical compositional of this nanowall obtained by means of energy dispersive X-ray (EDS) spectroscopy measurements. From the EDS map, the different chemical components of the NWs can be clearly distinguished: the MoS_2_ segment, embedded within the protective metal layer tungsten (W), and the silicon (Si) substrate.

**Figure 3:**
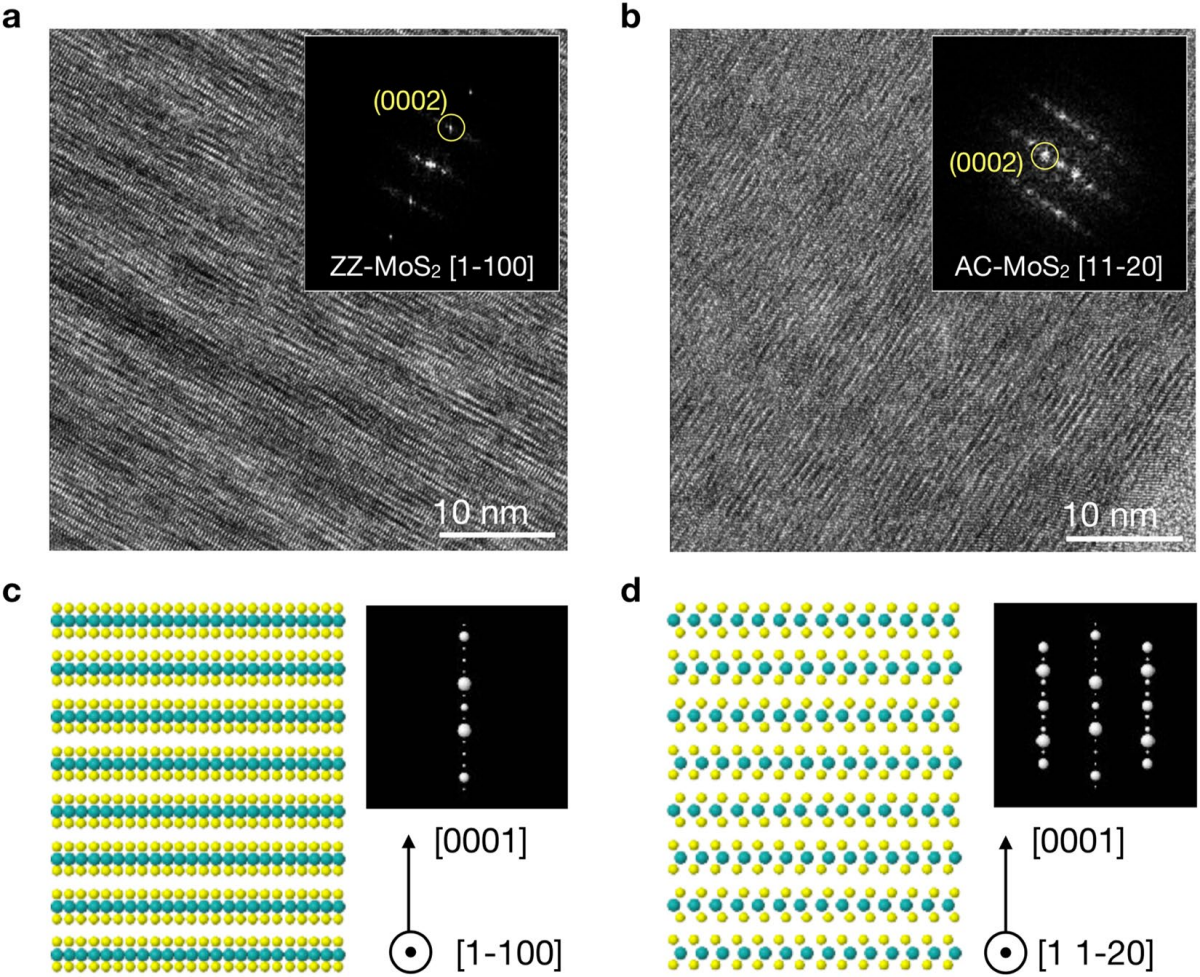
(**a**,**b**) HRTEM micrographs of representative ZZ and AC MoS_2_ nanowalls, respectively. The insets indicate the corresponding fast Fourier transform (FFT). **(c)** and **(b)** The atomic modelling associated to the AC and ZZ orientations of the NWs, together with the theoretical calculation of the expected FFTs.

From the crystalline structure studies carried out by means of high-resolution TEM measurements (Fig. 3), we are able to confirm the specific edge site configuration for the two NW arrays. Figure 3a,b display the results of the TEM measurements on the AC and ZZ MoS_2_ surfaces respectively. By comparing the two crystallographic orientations, AC and ZZ, we can observe the differences between the atomic arrangement of each surface, which are consequently characterized by different fast Fourier transforms (FFTs) (shown in the insets of Fig. 3a,b). From these results, it is clearly noticeable the excellent agreement between the experimental FFTs obtained from the TEM measurements and the corresponding ones calculated theoretically in terms of the expected atomic configuration (shown in Fig. 3c,d). These results provide direct confirmation that these vertically-oriented MoS_2_ nanowalls are in fact exposing ZZ and AC edge terminations, therefore validating our fabrication strategy.

### Fingerprinting the edge-type nature of MoS_2_ nanowalls

**Figure 4:**
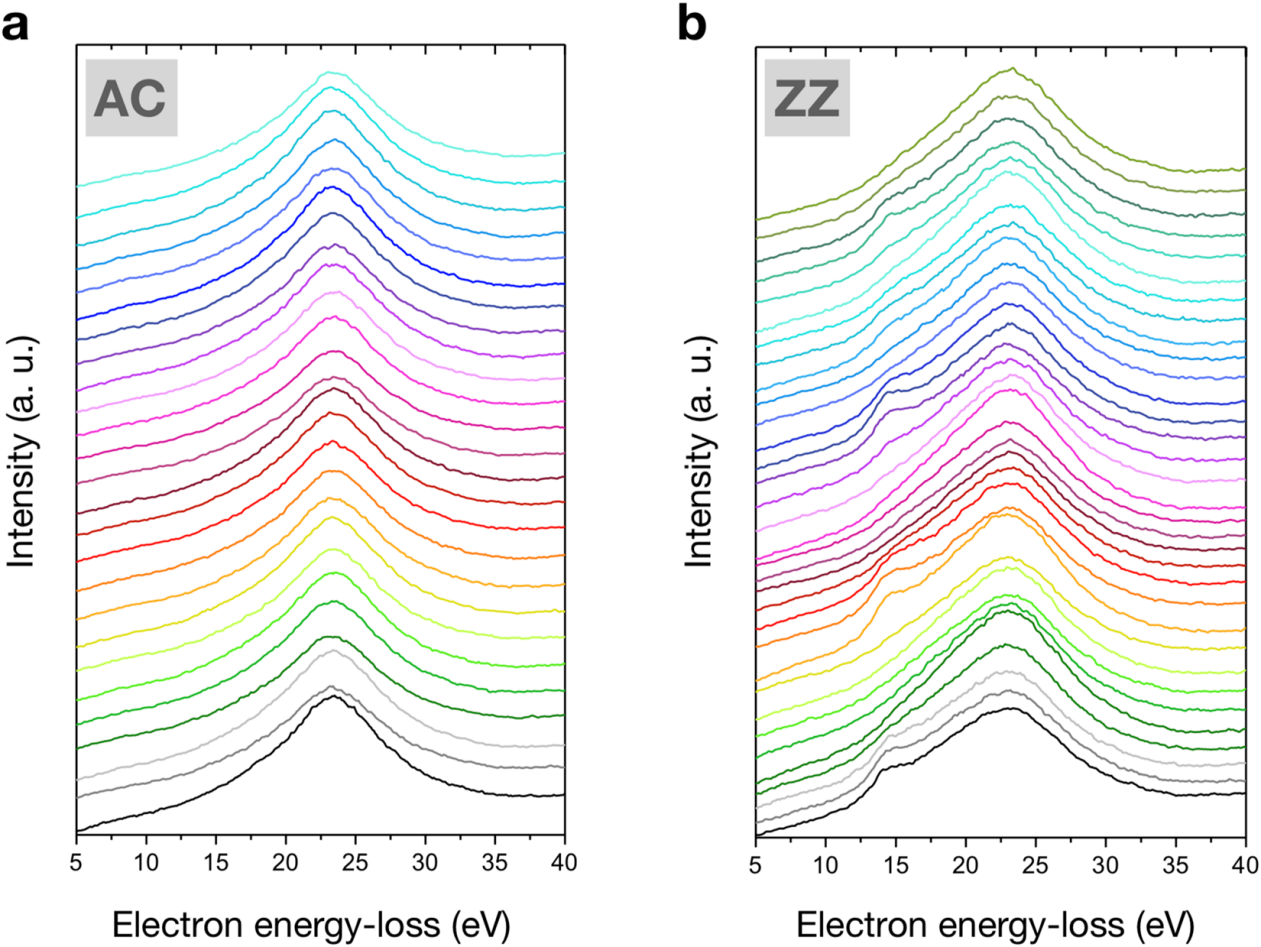
(**a**,**b**) EELS spectra taken at different positions of the AC and ZZ nanowalls, respectively, for the region of electron energy losses between 5 and 40 eV. Each curve corresponds to a different position along the NWs.

In order to pin down the local electronic properties of the AC and ZZ MoS_2_ NWs, electron energy-loss spectroscopy (EELS) measurements have been carried out in a scanning transmission electron microscope (STEM). In Fig. 4 we show the energy-loss spectra corresponding to both the AC and ZZ surfaces, taken at different points along the length of the nanowall. As it can be observed in the two sets of EELS spectra, the MoS_2_ bulk plasmon signal appears at 23.4 eV in both samples with similar intensities and general shape, in agreement with previous analyses^27,28^. Nevertheless, the MoS_2_ surface plasmon peak, present at 15.2 eV, turns out to appear only on a restricted subset of the spectra of the ZZ-nanowalls. Considering that the fabricated AC-nanowalls are thinner than the ZZ-terminated ones, the presence of the surface MoS_2_ plasmon on the ZZ-nanowalls cannot be attributed to a lower thickness of the sample. Therefore, the origin of this peak should be caused by another phenomenon.

In that respect, it is important to notice that the MoS_2_ surface plasmon peak appears and disappears in a periodic manner, depending on the specific position along the nanowall where the EELS spectrum is collected. It is found that the positions which correspond to local maxima of the intensity associated to this surface plasmon peak are separated by around 12 nm between each other. This behavior can be attributed to the presence of metallic surface plasmon polaritons (SPP), which are planar waves appearing at the interfaces between a metal and a dielectric material under some external excitation, such as an electron beam^29^. That could correspond to the oscillatory character present in our EELS spectra. Therefore, from this analysis, we can conclude that the ZZ MoS_2_ NWs surfaces present a clear metallic character. On the contrary, the AC MoS_2_ NWs do not exhibit such metallic behavior. With this result, we can hereby confirm that the ZZ MoS_2_ NWs are dominantly enclosed by zig-zag edges structures. It is also worth mentioning here that no signal arising from neither the metal layer nor the Ga used for the FIB milling were present at any of the acquired EELS spectra, indicating that the possible contamination from Ga in the nanowalls is non-existing.

## Discussion

In order to further validate the onset of the metallic behavior observed in the ZZ MoS_2_ nanowalls (NWs), we calculated the corresponding density of states (DOS) by means of *ab-initio* calculations in the framework of density functional theory (DFT). The van der Waals (vdW) interactions characteristic of MoS_2_ were incorporated by using the nonlocal vdW functional model^30^ as implemented in the WIEN2k code (see Methods for further details).

**Figure 5:**
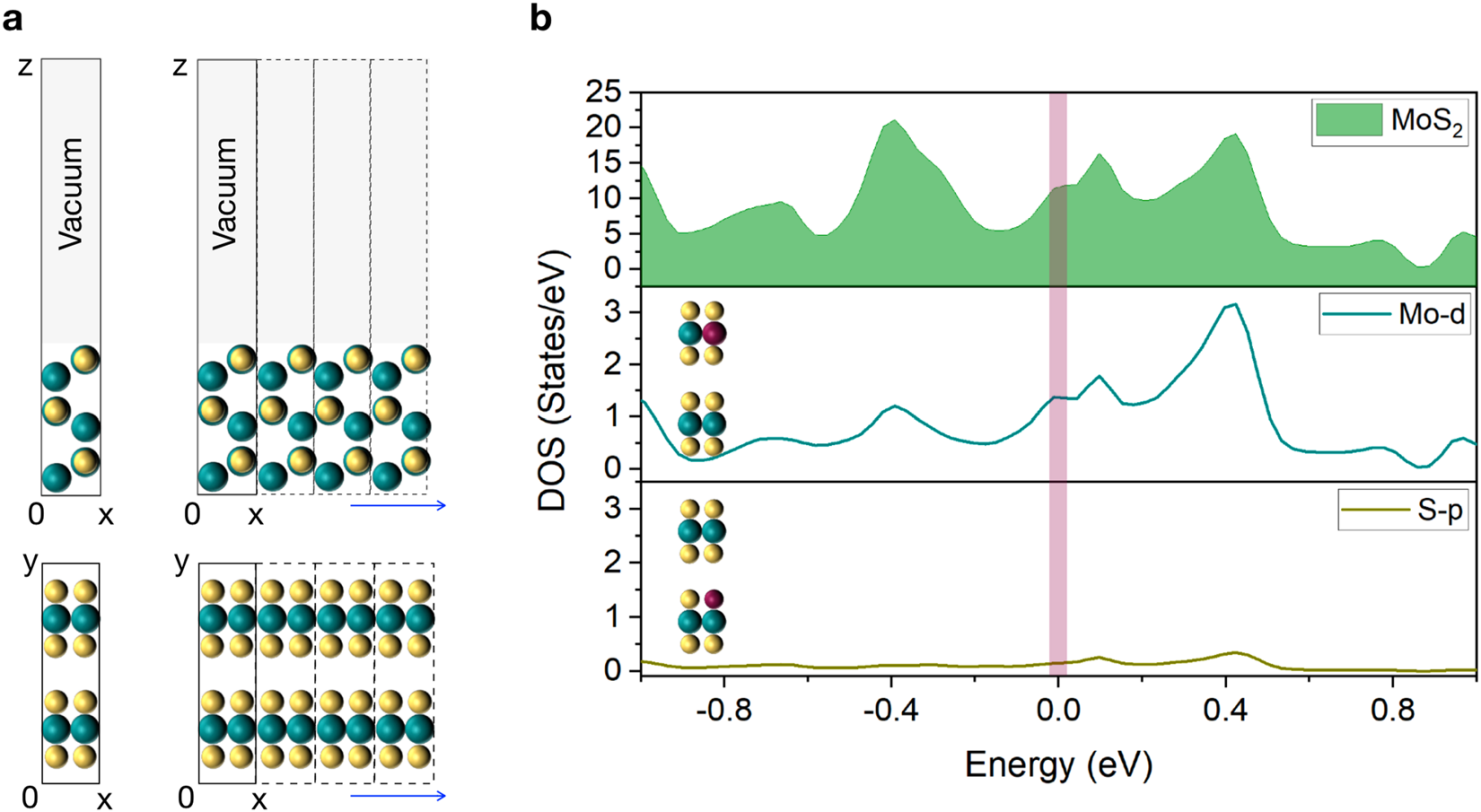
(**a**) The ZZ MoS_2_ nanowall can be modelled by constructing a 1 × 3 × 1 supercell with vacuum in the *z* direction. A vacuum layer with length of 17.170 Å was inserted along the ZZ edge of the nanowall, in order to to avoid spurious interactions between the repeating supercell images. This 1 × 3 × 1 supercell in the *x* and *z* directions was used to determine the density of states associated to the ZZ MoS_2_ nanowall, which can be treated as a two-dimensional sheet composed by ZZ nanorribons stacked along the *y* direction. (**b**) (*Top panel*) density of states of ZZ MoS_2_ nanowalls, (*central and bottom panels*) the individual contributions to the DOS from the Mo and S atoms located at the ZZ surface.

We modeled the ZZ MoS_2_ nanowall by constructing a 1 × 3 × 1 supercell of MoS_2_, as shown in Fig. 5a. In order to minimize the interactions between periodic images due to the 3D boundary conditions, we introduced a vacuum layer such that the distance between periodic images is 17.170 Å.

The resulting calculated total DOS for the ZZ MoS_2_ NW is displayed in Fig. 5b. A clear absence of a gap in the DOS near the Fermi energy is observed, which implies a finite probability (11.65 states/eV) for states just below and above the Fermi energy level being populated, highlighting the metallic behavior of the ZZ MoS_2_ NWs. Figure 5b (middle panel) also displays the individual contributions of the 4d states of Mo atoms located at the surface of the ZZ MoS_2_ nanowall. These 4d states of Mo are also observed to cross over the Fermi energy (1.35 states/eV), contributing therefore to the metallic character of the ZZ MoS_2_ nanowall. The individual contribution of the 3p states of S atoms located at the surface of the NW turns out to be much smaller from the DFT calculation, 0.08 states/eV. Therefore, the dominant contribution to the metallic character of ZZ MoS_2_ nanowalls can be confidently attributed to Mo-4d states of MoS_2_.

In this work, we have presented a novel approach for the top-down fabrication of ordered vertically-oriented MoS_2_ nanostructures (denoted as nanowalls) which makes possible to achieve at the same time, a large density of exposed active edge sites while also to controllably select whether these are of the AC or ZZ types. The crystallographic nature of the exposed surfaces has been validated by means of high-resolution TEM measurements. We have also studied the local electronic properties of these NW surfaces by means of EELS, finding direct evidence of the metallic character of the ZZ surfaces as indicated by the presence of MoS_2_ surface plasmon peak.

The metallic nature of the ZZ MoS_2_ nanowalls can be exploited to open new opportunities for nanoengineering the edge type in MoS_2_ nanostructures as well as in related layered materials. This would allow new exciting opportunities both for fundamental physics and technological applications in electronics, optoelectronics, photovoltaics, and photocatalysts.

## Methods

### Focus ion beam patterning for the fabrication of edge-controlled MoS_2_ nanowalls

MoS_2_ bulk crystal obtained from Alfa Aesar (99.999% purity) was mechanical exfoliated with Poly-Di-Methyl-Siloxaan (PDMS) and then transferred to a SiO_2_/Si substrate. The MoS_2_ nanostructures were milled using a FEI Helios G4 CX focus ion beam. The ion milling procedure was carried out using a very low energy electron beam of 15 kV, and an ion beam of 2 pA. Before the milling procedure was carried out, a protective metal (W) layer of 500 nm of thickness was deposited on top the selected areas.

### Characterization techniques

Transmission Electron Microscopy (TEM) measurements were carried out in a Titan Cube microscope using an acceleration voltage of 300 kV. Its spatial resolution at Scherzer defocus conditions is 0.08 nm in the High-Resolution Transmission Electron Microscopy (HRTEM) mode, whilst the resolution is around 0.19 nm in the HAADF-STEM (High Angle Annular Dark Field – Scanning Transmission Electron Microscopy) mode. Electron Energy Loss Spectroscopy (EELS) experiments were carried out using a Gatan Imaging Filter (GIF) spectrometer, employing a collection semi-angle of 2.95 mrad, a convergence semi-angle of 14 mrad, and an aperture of 2 mm. The energy resolution obtained by using these parameters in EELS was 0.9 eV, with an exposure time of 0.1 s/spectrum and an energy dispersion of 0.1 eV/channel.

### First-principle calculations

The density of states (DOS) calculations were performed using both linearized augmented plane wave (LAPW) and local orbitals (LO) methods implemented in the WIEN2k package^31^. The nonlocal van der Waals^32,33^ (vdW) interactions used for the DOS calculations uses optB88^34^ for the exchange term, the local density approximation^35^ (LDA) for the correlation term, and the DRSLL kernel for the non-local term^36^. For the non-local vdW integration the cut-off density r_c_ was set to 0.3 bohr^−3^, while the plane wave expansion cut-off G_max_ was set to 20 bohr^−1^. No spin polarization was considered. The lattice parameters were found by volume and force optimization of the supercell, such that the force on each atom was less than 1.0 mRy/bohr. The total energy convergence criteria was set to be 0.1 mRy between self-consistent field (SCF) cycles, while the charge convergence criteria was set to 0.001*e*, with *e* the elementary unit charge. The core and valence electron states were seperated by an energy gap of −6.0 Ry. Furthermore, the calculations used an R*k_max_ of 6.0, where *R* is the radius of the smallest Muffin Tin sphere, and *k*_*max*_ is the largest *k*-vector. The first Brillouin zone for the lattice parameter calculations was sampled with 100 *k*-points using the tetrahedon method of Blöchl *et al*.^37^, which corresponds to 21 k-points in the irreducible Brillouin zone. With the above parameters the optimized lattice parameters were *a* = 3.107 Å and *c* = 2.087 Å, which are in good agreement with the experimental values *a* = 3.161 Å and *c* = 12.295 Å^38^. The DOS was calculated with a denser *k*-point sampling of the Brillouin zone consisting of 1600 *k*-points, corresponding to 630 *k*-points in the irreducible Brillouin zone.

## Supplementary information

Supplementary Information
