# Supplementary material for: Metallic edge states in zig-zag vertically-oriented MoS2 nanowalls
Source: Sci Rep. 2019 Oct 30;9:15602. doi: 10.1038/s41598-019-52119-3 (PMC6821918; doi:10.1038/s41598-019-52119-3)
Supplement: Supplementary file 1 — Supplementary Information [file 41598_2019_52119_MOESM1_ESM.doc]

Supplementary Information:

Metallic edge states in zig-zag vertically-oriented MoS_2_ nanowalls

M. Tinoco^1,2,†^, L. Maduro^1,†^, and S. Conesa-Boj^1,*^

*^1^ Kavli Institute of Nanoscience, Delft University of Technology, 2628CJ Delft, the Netherlands*

*^2^ ICTS – Centro Nacional de Microscopía Electrónica, Universidad Complutense, 28040 Madrid, Spain*

***E-mail: [*s.conesaboj@tudelft.nl*](mailto:s.conesaboj@tudelft.nl)

^†^ Equal contribution.

**Section A. Top-down optimization of MoS_2_ nanowalls using focus ion beam.**

Gallium-based focus ion beam (FIB) milling was used to sculpt the MoS_2_ nanowalls. This method has been successfully applied for sculpting tailored nanostructures of different shapes and sizes in a wide variety of materials. [1, 2, 3]

|  |
| --- |
| **Figure SI-1. (a)** SEM image of the sculpted MoS_2_ flake. This flake is lying on a Si/SiO_2_ wafer; **(b)** Initial attempts for sculpting MoS_2_ nanowalls by means of FIB. |

|  |
| --- |
| **Figure SI-2. (a)** and **(b)** 0º and 52º tilted SEM images corresponding to the first milling step. **(c)** and **(d)** 0º and 52º tilted SEM images after milling the lateral of the nanowalls. The final MoS_2_ nanowalls are displayed in **(e)** and **(f)**, which correspond to the SEM images 0º and 52º tilted, respectively. The scale bar of the all SEM images from the left panels is 1 μm, while that from the right panels is 500 nm. |

**Figure SI-1a** displays a scanning electron microscopy (SEM) image of the MoS_2_ flake used for the top-down nanowall fabrication. This MoS_2_ flake was first deposited on a Si/SiO_2_ wafer. Subsequently, the MoS_2_ flake was directly exposed to the ion beam,

see **Figure SI-1b**, in a region far away from the interesting area. In these procedures, both the voltage and current of the electron beam were fixed to 15 kV and 2pA, respectively. In the areas indicated as A, B and C in **Figure SI-1b**, three solid rectangles separated by 50, 80 and 100 nm, respectively were used for the milling. The dimensions of the rectangle used were 1 x 0.2 x 1 µm. For regions D and E, a cleaning pattern was used instead, which in region D was going upwards and in E downwards. The dimensions of the cleaning pattern were in this case 1 x 0.2 x 1 µm.

Based on previous studies, we decided to use the cleaning function for patterning the active surface of the nanowalls, in order to avoid any damage, while the laterals of the nanowalls were removed by employing a milling function. However, a repeated deposition of MoS_2_ on top of the nanowalls during their creation represented an issue (**Figure SI-2a**). Several cleaning steps were applied to get rid of this re-deposition to re-shape again the MoS_2_ nanowalls to the desired morphology. Thanks to this successive milling and cleaning processes, it was possible to reduce the size of the nanowalls to the preferred dimensions (**Figure SI-2b**). Unfortunately, after successive milling and cleaning most of the MoS_2_ was milled away.

|  |
| --- |
| **Figure SI-3. (a)** and **(b)** SEM images of the final MoS_2_ nanowalls by using a W metal layer to protect the MoS_2_ during milling process. The Scale bar in **(a)** and **(b)** are 1µm**.** |

*W-assisted MoS_2_ nanowalls fabrication*

In order to avoid the milling of the upper part of the MoS_2_ nanowalls, a protective W layer of 500 nm of thickness was deposited on top of the MoS_2_. By using this protective layer, thinner MoS_2_ nanowalls were achieved, with a resulting thickness of around 65 nm approximately, see **Figure SI-3**.

**References**

[1] Friedensen, S., Mlack, J. T & Drndic, M. Materials analysis and focused ion beam nanofabrication of topological insulator Bi_2_Se_3_ *Sci. Rep* **7**, 13466 (2017)

[2] Bhattacharyya, B., Sharma, A., Sinha, B., Shah, K., Jejurikar, S., Senguttuvan, T. D. & Husale S. *Sci. Rep* **7**, 7825 (2017)
